# Streamlining Apheresis: A Dual‐Intervention Quality Improvement Initiative to Increase the Efficiency in Stem Cell Collection

**DOI:** 10.1002/jca.70066

**Published:** 2025-10-23

**Authors:** Ivy E. Verriet, Renee Dickey, Sue Sinclair, Karla Schebesch, Shona Philip, Anargyros Xenocostas, Uday Deotare

**Affiliations:** ^1^ Department of Medicine, Schulich School of Medicine and Dentistry Western University London Canada; ^2^ Blood and Marrow Transplant Program, London Health Sciences Centre London Canada; ^3^ Division of Hematology, Department of Medicine, Schulich School of Medicine and Dentistry Western University London Canada; ^4^ The Centre for Quality, Innovation and Safety, Department of Medicine, Schulich School of Medicine and Dentistry Western University London Canada

**Keywords:** apheresis, autologous stem cell transplant, CD34+ cell collection target, quality improvement

## Abstract

Autologous stem cell transplantation (ASCT) requires efficient collection of peripheral blood stem cells. At London Health Sciences Centre (LHSC), high‐risk multiple myeloma patients are routinely booked for three‐day apheresis collections to meet higher CD34+ cell count targets, though many do not require all scheduled days, leading to resource inefficiencies. A quality improvement initiative was implemented to reduce unnecessary apheresis sessions through two interventions: (1) lowering CD34+ cell count target thresholds (from 6 × 10^6^ to 5 × 10^6^ cells/kg for tandem collections and from 3 × 10^6^ to 2.5 × 10^6^ for single collections), and (2) increasing total blood volume (TBV) processed from 3× to 4× for patients within certain target thresholds. Two Plan‐Do‐Study‐Act (PDSA) cycles were conducted between March 2024 and March 2025 involving 76 patients. Outcome measures included collection days saved and cost savings, and post‐transplant engraftment times served as a balancing measure. A total of 39.4% of patients avoided at least one collection day due to these interventions. Third‐day collection usage in high‐risk myeloma patients decreased from 25% to 5.9%. Mean collection days fell significantly in this group (2.21–1.8; *p* = 0.0015), with total cost savings of CAD $72 734.97. No significant differences were observed in neutrophil or platelet engraftment times, confirming preserved clinical efficacy. Implementing lower CD34+ cell count targets and increased TBV processing significantly reduced apheresis sessions and costs without compromising engraftment outcomes. These changes have become the standard of care at LHSC and may serve as a feasible model for other transplant centers.

## 1 Introduction

Autologous stem cell transplant (ASCT) is a well‐established treatment indicated for several hematologic malignancies, including lymphoma and multiple myeloma [1]. ASCT involves the collection of hematopoietic stem cells (HSCs) from the patient, who serves as both the donor and recipient. To facilitate collection, patients receive granulocyte colony‐stimulating factor (G‐CSF) with or without a CXCR4 antagonist (e.g., Plerixafor) to mobilize HSCs from bone marrow into the peripheral blood [1].

At London Health Sciences Centre (LHSC), collection targets for standard‐risk multiple myeloma and lymphoma patients are set at 3 × 10^6^ CD34+ cells/kg, while high‐risk multiple myeloma patients require 6 × 10^6^ CD34+ cells/kg to allow for tandem transplantation. These targets align with published guidelines, where a minimum of 2 × 10^6^ CD34+ cells is considered necessary for successful engraftment [2, 3]. There is a consensus that higher target doses may result in faster engraftment times, but there must be consideration for the balance between target values and the number of apheresis sessions required to obtain target collection [3]. At LHSC, the scheduling of collection days varies by indication, with high‐risk multiple myeloma patients automatically booked for 3 days of apheresis to accommodate their higher target dose. All other indications are scheduled for 2 days of apheresis.

During collection, the patient's blood volume is typically processed three times, after which a CD34+ cell count is performed on the collected blood to assess if the target has been met. If the target is not achieved, the patient receives additional doses of Filgrastim and/or Plerixafor and returns for another collection the following day. This process can continue for up to 3–4 days until the required number of CD34+ cells is collected. At the end of each collection session, the harvested HSCs undergo cryopreservation and are aliquoted for long‐term storage.

A retrospective review of our institution's (LHSC) collection data revealed that 75% of third‐day collection appointments for high‐risk multiple myeloma patients were unused, leading to inefficient resource allocation and limiting the capacity to schedule additional patients. Moreover, each additional collection day results in additional aliquoted samples, leading to reduced storage capacity and resource inefficiencies within the processing facility. To optimize the efficiency of the apheresis process while maintaining high standards of care, we implemented a quality improvement (QI) initiative aimed at reducing unnecessary collection days and improving workflow efficiency.

## 2 Materials and Methods

### 2.1 Baseline Information

At our institution, the existing mobilization protocol was as follows: all patients received Filgrastim (10 mcg/kg daily) for 4 days prior to collection. On the morning of Day 1, peripheral blood was analyzed by flow cytometry to measure circulating CD34+ cells. Two criteria were used to determine whether to proceed with collection. If the absolute CD34+ cell count was less than 10 × 10^6^ cells/L, collection was postponed regardless of diagnosis. If the predicted yield, based on circulating CD34+ cell levels, patient weight, and expected blood volume processed, was below the predefined collection target (2.5–3.0 × 10^6^ or 5.0–6.0 × 10^6^ CD34+ cells/kg depending on transplant type), collection was proceeded but with plan for next day apheresis. In either case, patients received Plerixafor and Filgrastim, and collection was resumed the following day after re‐evaluation. After each apheresis session, CD34+ counts from the product were used to assess adequacy. If the collected product was below 50% of the target, patients received Filgrastim and Plerixafor and returned for further collection the next day. This cycle was repeated until the target was achieved or the patient was deemed unable to proceed further.

### 2.2 Baseline Data

To establish baseline measures, a retrospective analysis was conducted to evaluate the average number of apheresis collection days by diagnosis over a three‐year period (2021–2024). The mean number of collection days was 2.00 ± 0.77 (*n* = 18) for standard‐risk multiple myeloma, 2.21 ± 0.68 (*n* = 104) for high‐risk multiple myeloma, and 1.53 ± 0.56 (*n* = 64) for lymphoma. Additionally, the frequency of third‐day collection was assessed: 27.8% (5 of 18) of standard‐risk multiple myeloma patients, 25.0% (26 of 104) of high‐risk multiple myeloma patients, and 3.1% (2 of 64) of lymphoma patients required a third day of collection.

### 2.3 Quality Improvement Interventions

**FIGURE 1:**
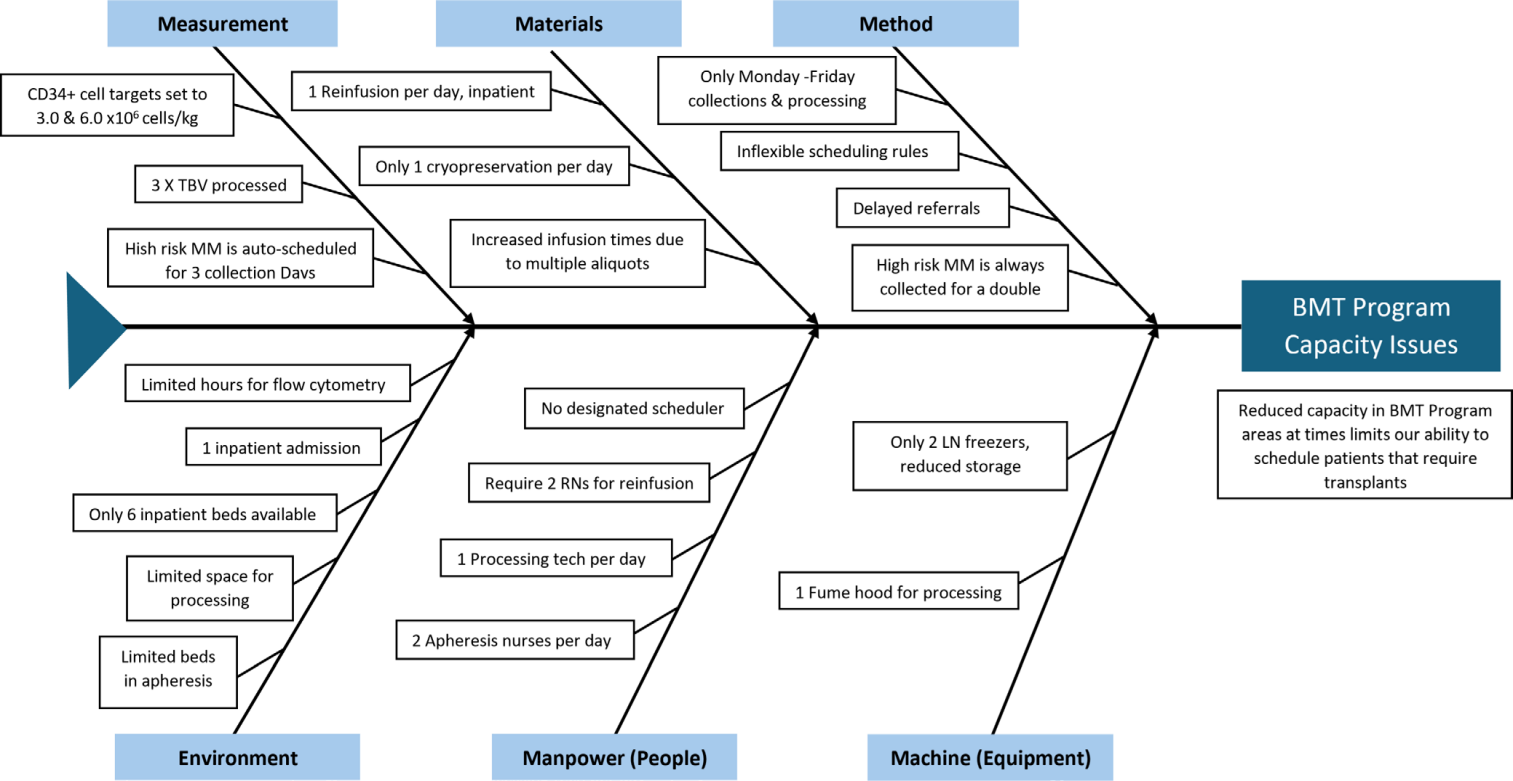
Ishikawa Fishbone diagram identifying barriers to efficient stem cell collection at the Bone Marrow Transplant (BMT) program. Root causes leading to capacity issues are categorized under six domains: Method, Materials, Measurement, Environment, Manpower (People), and Machine (Equipment). MM: multiple myeloma, RN: Registered nurse, LN: liquid nitrogen.

After identifying the need to reduce the number of collection days, a cause‐effect analysis and Ishikawa fishbone diagram were generated (Figure 1). The root causes identified included human resources (nursing and processing technicians), a restricted number of apheresis unit beds, inadequate freezer storage for aliquoted stem cell products, and inefficiencies in target‐setting and scheduling for high‐risk multiple myeloma patients.

The Plan‐Do‐Study‐Act (PDSA) framework was utilized to evaluate and reassess potential interventions. The interventions that were considered included reducing CD34+ cell collection targets, increasing total blood volume (TBV) processed during apheresis, expanding physical infrastructure (e.g., additional apheresis beds and machines), and increasing staffing. However, infrastructure expansion and hiring additional staff would require administrative approval and substantial costs that were impractical for the scope of this project.

Ultimately, reducing CD34+ cell targets and increasing TBV processing were selected as the primary interventions. These strategies required no additional cost, were immediately implementable, and were expected to improve unit capacity and patient experience as well as reduce demand for apheresis resources. Two PDSA cycles were implemented to determine the impact of these two key changes.

In the first PDSA cycle, the first intervention was reducing the target for CD34+ cells. For single collections, the target would be reduced from 3 × 10^6^ to 2.5 × 10^6^ CD34+ cells/kg, and for a double collection, the target would be reduced from 6 × 10^6^ to 5 × 10^6^ CD34+ cells/kg. The second intervention was to increase the TBV processed. For patients whose predicted CD34+ cell collection was within 0.5 × 10^6^ + cells/kg of the target, TBV processing was increased from 3× TBV to 4×. The first cycle aimed to reduce the number of collection days by at least 1 day for 30% of patients in the apheresis unit.

In the second PDSA cycle, to increase the impact of our intervention, we expanded the range for which 4× TBV processing would be applied. The threshold was increased from 0.5 × 10^6^ CD34+ cells/kg to 1.0 × 10^6^ CD34+ cells/kg within the target. This modification allowed a greater proportion of patients to undergo increased TBV processing, thereby increasing the likelihood of achieving the target in fewer sessions. The decreased CD34+ cell target was maintained throughout the second cycle.

Prior to undergoing autologous stem cell collection, all patients at our center routinely receive comprehensive cardiac and renal assessments, including a two‐dimensional echocardiogram, evaluation of renal function via creatinine clearance calculation, and electrolyte testing, including calcium levels. Calcium supplementation is provided based on clinical symptomatology. These standard pre‐collection assessments were maintained consistently across all patients, including those who underwent increased total blood volume (TBV) processing.

### 2.4 Study Population

All patients who underwent ASCT at LHSC between March 2024 and March 2025 were included in the study. The first PDSA cycle ran from March to September 2024, and the second PDSA cycle ran from October 2024 to March 2025. A total of 76 patients met the inclusion criteria, 42 in the first cycle and 34 in the second cycle, representing the following diagnostic categories: high‐risk multiple myeloma, standard‐risk multiple myeloma, or lymphoma.

### 2.5 Family of Measures

The primary outcome measure was the mean number of collection days required per patient, stratified by diagnosis and PDSA cycle, to evaluate changes following the intervention. Secondary outcome measures included collection days saved, defined as the reduction in apheresis sessions needed to meet the CD34+ cell targets, and estimated cost savings associated with reduced collection days. The process measure was the proportion of patients requiring a third collection day, used to assess whether interventions were successfully implemented and sustained across each PDSA cycle. The balancing measure was time (days) to neutrophil (ANC) and platelet engraftment post‐transplant.

#### 2.5.1 Primary Outcome Measure

The mean number of collection days required per patient was calculated and stratified by diagnosis and PDSA cycle. Comparisons were made between baseline and each PDSA cycle, and the combined effect of both cycles was evaluated to determine the overall impact on collection days across the study population.

#### 2.5.2 Secondary Outcome Measures

To assess the impact of reducing the CD34+ cell target, a collection day was considered saved if a patient met the final required count of 2.5 × 10^6^ to 3.0 × 10^6^ CD34+ cells/kg for a single transplant or 5.0 × 10^6^ to 6.0 × 10^6^ CD34+ cells/kg for a double transplant. These patients would have otherwise undergone an additional collection day under the pre‐intervention protocol.

To evaluate the effect of increasing TBV processing from 3× to 4×, we estimated the number of CD34+ cells that would have been collected under the pre‐intervention protocol using the formula: (Total CD34+ cells collected ÷ 4) × 3. Patients who underwent 4× TBV processing and achieved a final CD34+ cell count between 3.0 × 10^6^ and 3.3 × 10^6^ cells/kg for a single collection or between 6.0 × 10^6^ and 6.6 × 10^6^ cells/kg for a double collection would have been below the new minimum target (2.5 × 10^6^ or 5.0 × 10^6^ cells/kg) if they had undergone 3× TBV processing, according to the formula. Therefore, their ability to meet collection goals in fewer days was attributed to the increased TBV rather than the lowered target threshold.

Patients who underwent 4× TBV processing and achieved a final CD34+ cell volume between 2.5 × 10^6^ and 3.0 × 10^6^ for a single transplant or 5.0 × 10^6^ and 6.0 × 10^6^ for a double transplant were considered to have saved at least one collection day due to the combination of both interventions.

Cost saved was assessed according to the amount of materials and services that would have been used on each collection day saved per patient. Patients who did not need to return for an additional day due to an intervention saved additional Filgrastim and/or Plerixafor injections, the cost of collection supplies, and the cost of staffing a nurse and processing technician. Costs saved were assessed on a per patient case.

#### 2.5.3 Process and Balancing Measures

To assess the proportion of third‐day collections used, analysis was limited to patients with high‐risk multiple myeloma, as they were the only group automatically scheduled for three‐day collections. This proportion was calculated for each PDSA cycle individually, as well as for both cycles combined.

As a balancing measure, we monitored the number of days from transplant to ANC and platelet engraftment to assess whether our interventions impacted engraftment quality, comparing affected and unaffected patients across both cycles.

### 2.6 Statistical Analysis

Comparisons of mean collection days between pre‐ and post‐intervention groups were performed using post hoc Mann–Whitney *U* tests for non‐parametric data, with Bonferroni correction applied to adjust for multiple pairwise comparisons across the baseline, PDSA cycle 1, and PDSA cycle 2 groups. Chi‐square testing was used to assess differences in third‐day collection use. Cost analysis was conducted by calculating per‐patient savings in staffing, mobilization agents, and collection expenses.

One‐way ANOVA with Welch's correction for unequal sample sizes was performed to compare mean collection efficiencies between the three groups: baseline, PDSA cycle 1, and PDSA cycle 2.

To assess baseline measures, we compared the mean value of days post‐transplant to ANC and platelet engraftment between patients affected and unaffected by the interventions, using a Mann–Whitney *U* test for non‐parametric data. Patients who did not live to transplant were excluded. Statistical significance was defined as a *p*‐value < 0.05 for all statistical analyses.

## 3 Results

**FIGURE 2:**
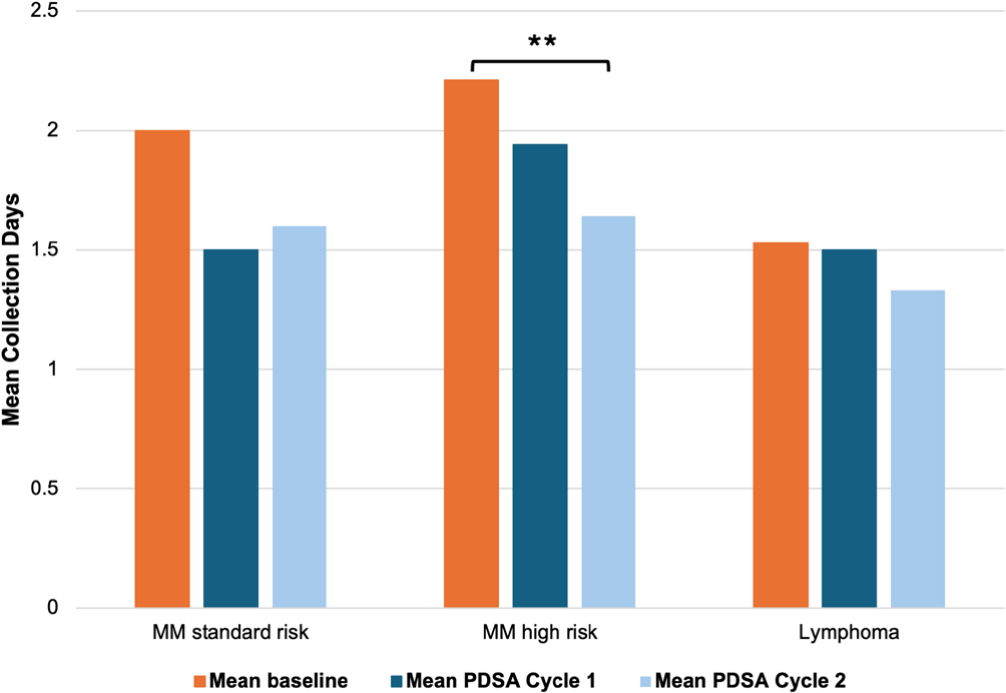
Reduction in mean collection days following intervention, stratified by diagnosis and cycle. Baseline represents patients from 2021 to March 2024. **p < 0.005. MM; multiple myeloma, PDSA; Plan‐Do‐Study‐Act.

The mean number of collection days after the first PDSA cycle was 1.50 ± 0.53 (*n* = 8) for standard‐risk multiple myeloma, 1.94 ± 0.54 (*n* = 18) for high‐risk multiple myeloma, and 1.50 ± 0.52 (*n* = 16) for lymphoma. Following the second cycle, means were 1.60 ± 0.55 (*n* = 5), 1.65 ± 0.61 (*n* = 17), and 1.33 ± 0.49 (*n* = 12) for standard‐risk myeloma, high‐risk myeloma, and lymphoma, respectively. When data from both cycles were combined, the mean number of collection days for high‐risk multiple myeloma patients significantly decreased from 2.21 ± 0.68 pre‐intervention to 1.80 ± 0.58 (*n* = 35; *p* < 0.01). Reductions were also observed for standard‐risk myeloma from 2.00 ± 0.77 to 1.54 ± 0.52 (*n* = 13; *p* = 0.0924) and lymphoma from 1.53 ± 0.56 to 1.43 ± 0.50 (*n* = 28; *p* = 0.4236), though these changes were not statistically significant. Notably, the reduction in high‐risk myeloma collection days between cycle 1 and cycle 2 (1.94 ± 0.54 to 1.65 ± 0.61) was also significant (*p* < 0.01). While the trends in standard‐risk myeloma and lymphoma were not significant, they consistently demonstrated a decrease from baseline values (Figure 2).

**FIGURE 3:**
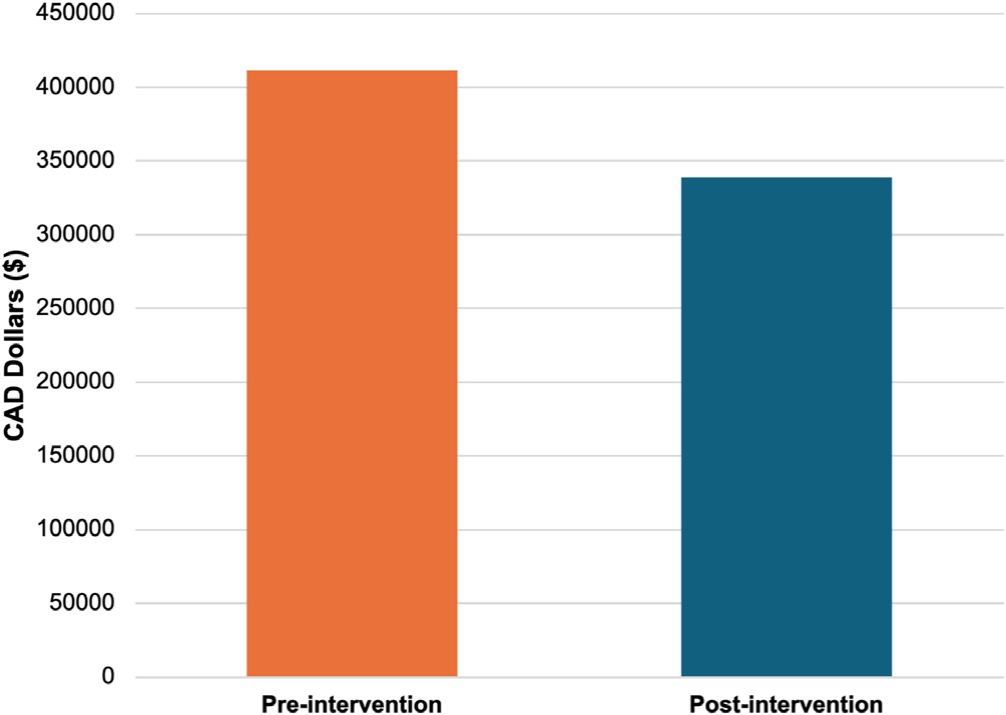
Comparison of cost before and after intervention. Pre‐intervention represents total cost spent if no collection days were saved for all patients between March 2024 and March 2025. Post‐intervention represents total cost spent for all patients between March 2024 and March 2025. CAD; Canadian Dollar.

The total cost savings attributed to the reduction in collection days amounted to CAD $72 734.97. On average, each collection day saved resulted in a cost reduction of $2424.499 per patient affected by our interventions (Figure 3). A detailed breakdown of cost savings is provided in Table 1.

**TABLE 1:** Cost savings breakdown.

Item/service	Cost per unit (CAD $)	Total cost saved (CAD $)
Filgrastim		
300 mcg	176.13	14463.70
480 mcg	218.81	
Plerixafor		
Price/mg	91.67	20885.97
Nursing administration	112.00	
Processing technician ($/hour)	50.24	10299.20
Nursing ($/hour)	56.00	12600.00
Collection and blood testing supplies^a^	482.87	14486.10
Total		**72734.97**

Abbreviation: CAD, Canadian Dollar.

^a^ Collection and blood testing supplies includes Optia collection set, acid‐citrate‐dextrose solution A (ACD‐A), saline solution, Tego connectors, calcium gluconate, blood warmer tubing, Y connectors, as well as blood tests (CBC, electrolytes, albumin, calcium) and flow cytometry.

Among the 76 patients included in the study, 39.4% (30 patients) saved at least one collection day due to the interventions. Of these, 33.3% (10 patients) met their target solely due to the lower CD34+ cell threshold, while 23.3% (7 patients) benefited solely from increased TBV processing, and 43.3% (13 patients) experienced the combined effect of both interventions. The breakdown for each PDSA cycle is detailed in Table 2.

**TABLE 2:** Distribution of patients by intervention and PDSA cycle.

	Reduced CD34+ target	Increased TBV processing	Both interventions	Did not qualify for interventions	Total patients
Cycle 1	6	6	6	24	42
Cycle 2	4	1	7	22	34

Abbreviations: PDSA, Plan‐Do‐Study‐Act; TBV, total blood volume.

**FIGURE 4:**
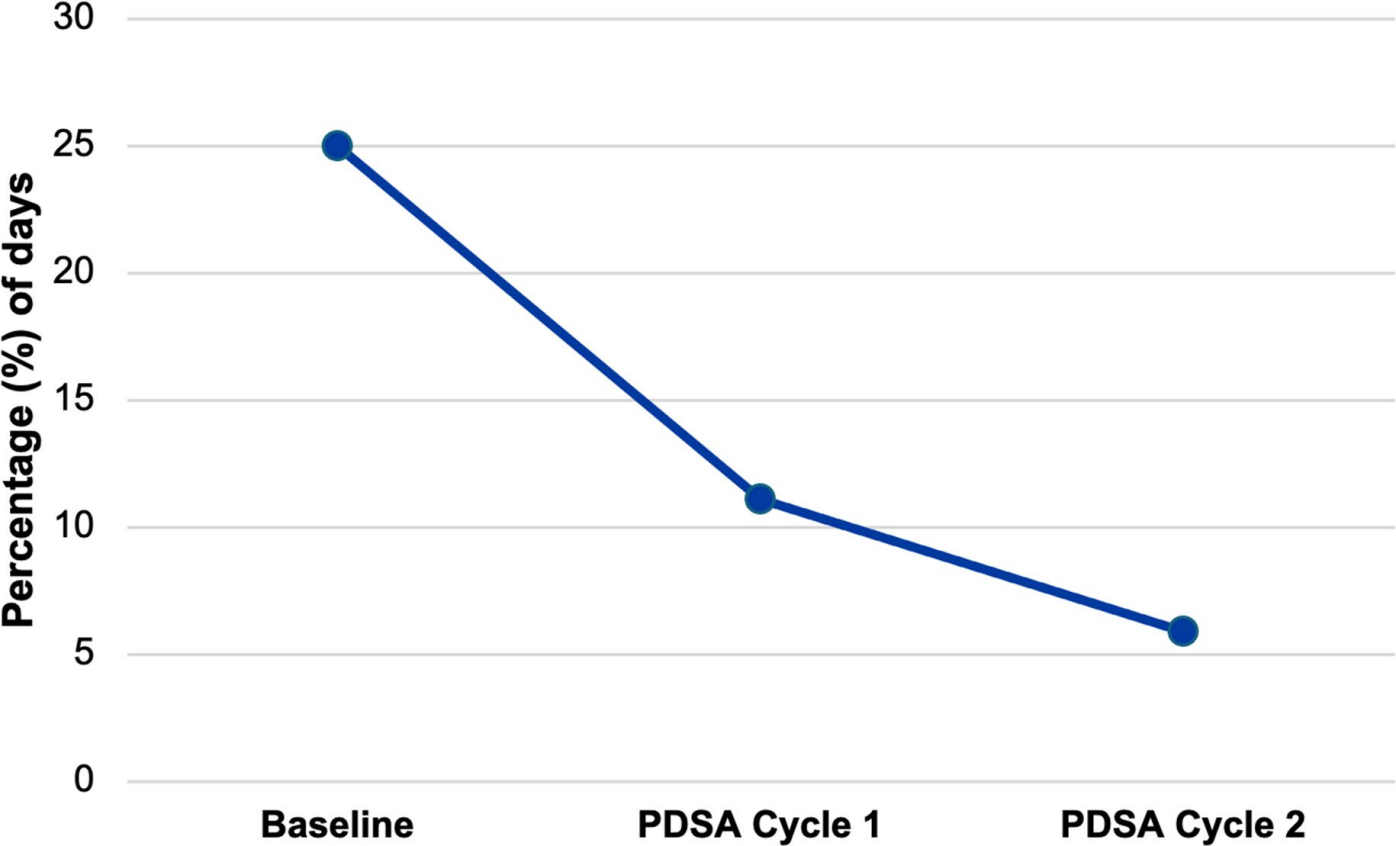
Percentage of third collection days used by high‐risk multiple myeloma patients stratified by PDSA cycle. Baseline represents patients from 2021 to March 2024 (pre‐intervention). PDSA; Plan‐Do‐Study‐Act.

The proportion of high‐risk multiple myeloma patients utilizing a third collection day decreased from 25% (26 of 104) pre‐intervention to 11.0% (2 of 18) after the first PDSA cycle and further decreased to 5.9% (1 of 17) following the second cycle (Figure 4). When considering both cycles together, the proportion of third‐day collections used declined significantly to 8.6% (*p* = 0.039). Additionally, aside from requiring an additional day to meet collection targets, no patients—regardless of diagnoses—failed to achieve their CD34+ cell count goals at any point during the study.

**FIGURE 5:**
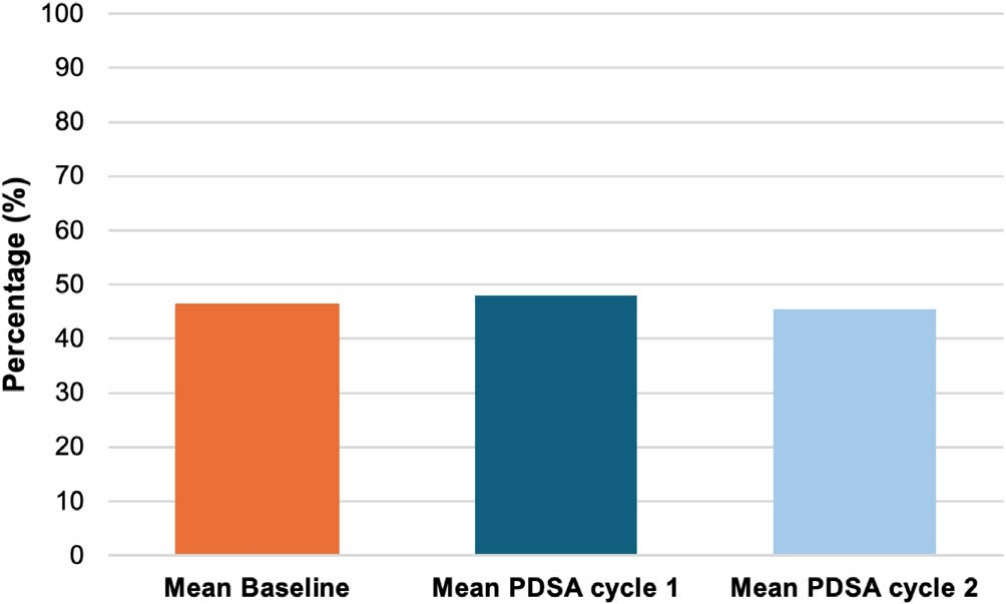
Comparison of collection efficiencies over cycles. Baseline represents patients from 2021 to March 2024 (pre‐intervention). PDSA: Plan‐Do‐Study‐Act.

Collection efficiency did not change over the course of the project. The mean collection efficiency was 46% ± 10% at baseline (*n* = 433), 48% ± 9% after PDSA cycle 1 (*n* = 72), and 45% ± 7% after PDSA cycle 2 (*n* = 65). There was no statistical significance between these means (*p* = 0.23) (Figure 5).

**FIGURE 6:**
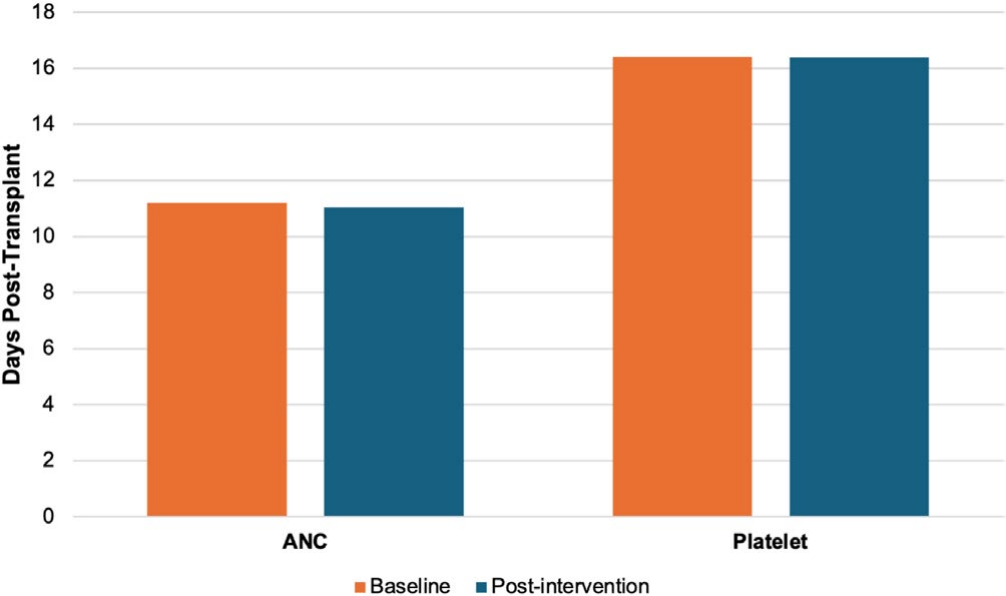
Comparison of ANC and platelet engraftment time between patients at baseline and post‐intervention. Baseline represents patients that were not impacted by interventions between March 2024 and March 2025. Post‐intervention represents patients that were impacted by interventions between March 2024 and March 2025. ANC; Absolute neutrophil count.

Importantly, balancing measures confirmed that there was no significant difference in mean time for ANC or platelet engraftment post‐transplant (*p* = 0.936 and 0.478, respectively), indicating that the quality of collected HSCs remained unaffected by interventions (Figure 6).

## 4 Discussion

This quality improvement initiative aimed to enhance the efficiency of autologous HSC apheresis collection at LHSC while maintaining the quality of care. Through two key interventions—reducing the CD34+ cell count target and increasing total blood volume (TBV) processing—our institution was able to significantly reduce the mean collection days for high‐risk multiple myeloma patients from 2.21 ± 0.68 to 1.8 ± 0.58 days (*p* < 0.01). As well, mean collection days for standard‐risk myeloma and lymphoma patients also trended down with the implementation of our interventions. Overall, we were able to reduce the use of third day collections from 25% pre‐intervention to 5.9% after the second PDSA cycle.

By decreasing the need for third‐day collections, we created additional availability in the apheresis unit, allowing more patients to be scheduled. With only two available collection slots per day, reducing unnecessary third‐day bookings allows more patients to access apheresis services without requiring significant additional resources. This change has been adopted as a permanent practice at our center, with all patients now scheduled for two collection days regardless of diagnosis. While this restructuring has the potential to optimize workflow and alleviate prior capacity constraints, ongoing nursing shortages at the time of publication have limited our ability to increase overall booking frequency; thus, the impact of this change is yet to be fully realized. No formal feedback survey was performed; however, informal feedback from nursing and technical staff has been positive, noting increased satisfaction due to fewer overall working days, despite longer individual collection days. Similarly, patient satisfaction has been informally observed to have improved, as they are undergoing fewer collection days.

From a financial perspective, reducing collection days has resulted in substantial cost savings of CAD $72 734.97 over 12 months. As well, each additional collection day necessitates the aliquoting and cryopreservation of four additional bags, increasing resource use and freezer storage demands. By minimizing third‐day collections, we reduced expenses associated with additional cryopreservation bags and freed up valuable storage space. As well, each collection day saved reduces the use of flow cytometry reagents required for pre‐collection CD34+ cell count assessment. Although these savings on freezer storage, cryopreservation supplies, and flow cytometry reagents were not formally calculated as part of our analysis, they represent meaningful reductions in facility resource utilization. Moreover, a reduction in collection days has directly reduced costs, including the reduced need for the administration of G‐CSF (Filgrastim) and CXCR4 antagonist (Plerixafor), decreased staffing costs, and reduced collection and laboratory processing expenses.

Patients have also directly benefited from these interventions. On average, patients now require fewer days of apheresis, regardless of diagnosis. This translates to lower financial burdens, as patients who previously required an additional collection day now save on accommodation, food, and transportation costs. For 43% of patients in our study, at least one fewer day was spent in the hospital, reducing the emotional and physical stress associated with prolonged treatment. These benefits enhance the overall patient experience and improve quality of life during the apheresis process.

Balancing measures were carefully evaluated to ensure that reducing collection days did not compromise patient outcomes. Engraftment rates for platelet and absolute neutrophil count (ANC) remained stable, with no significant differences observed for those affected or unaffected by intervention. This confirms that optimizing collection strategies did not negatively impact the quality of collected HSCs or patient recovery.

Many studies have focused on optimizing HSC mobilization for apheresis; however, most of these have investigated improvements in myeloid‐growth factor regimens. Studies that have examined CD34+ cell count targets have primarily focused on establishing a minimum, with evidence showing that targets below 2.5 × 10^6^ CD34+ cells/kg are associated with significant delays in neutrophil and platelet engraftment [3]. While some studies have attempted to define an ideal target, reporting that CD34+ cell count targets above 10 × 10^6^ CD34+ cells/kg result in earlier neutrophil engraftment by 1–2 days and earlier platelet engraftment by 2–4 days, these studies are not well controlled and do not fully account for trade‐offs involved in additional apheresis sessions [4, 5, 6]. Our study is unique in that our primary outcome measure focuses on collection days rather than solely on cell count, allowing us to evaluate how lowering CD34+ cell count targets impacts patient scheduling, cost savings, and efficiency while maintaining acceptable engraftment outcomes, as assessed by ANC and platelet engraftment times.

Additionally, a process known as large‐volume leukapheresis (LVL) which typically involves running a patient's blood volume 4 or more times has been previously shown in literature to enhance CD34+ cell yield per apheresis session [3]. Aside from increased blood processing, LVL processing also requires increased blood flow rate and the use of additional anticoagulants. This study is the first to our knowledge to demonstrate that a simple increase in TBV from 3× to 4× can have a meaningful impact on CD34+ cell collection without the need for additional supplies or changes in protocol. Moreover, a prior retrospective chart review examining the effect of TBV on patient outcomes found that higher apheresis volumes were associated with a significantly decreased risk of relapse and improved relapse‐free survival [7]. Our study builds upon this by showing that increasing TBV processing can reduce the number of collection days required while maintaining HSC yield, ultimately benefiting both the institution and the patient.

Importantly, these interventions have been implemented as the new standard of care at LHSC, demonstrating their long‐term feasibility and sustainability. Since the conclusion of our study, both changes have remained in place, ensuring ongoing efficiency improvements within the apheresis unit. These results may encourage other transplant centers to adopt lower CD34+ cell count targets and increase TBV processing to optimize the number of patients that can be accommodated while reducing costs and maintaining high‐quality engraftment outcomes.

## 5 Limitations

This study has several limitations. First, the estimation of collection days saved due to increased TBV processing relied on a linear formula—(Total CD34+ cells collected ÷ 4) × 3—to approximate what would have been collected under the previous protocol. This is not an exact measure, as individual variability in HSC mobilization may introduce some level of inaccuracy. Additionally, confounding factors such as differences in mobilization regimens, prior chemotherapy exposure, and patient‐specific characteristics (e.g., age, baseline CD34+ cell counts) could have influenced the results. Mean collection days used were stratified by diagnosis to help account for some of these confounding variables. Additionally, because of the nature of this quality improvement project, detailed demographic information such as age and sex was not collected. Future studies incorporating these variables could provide a more comprehensive analysis of factors influencing collection outcomes.

Second, the relatively small sample sizes within diagnostic subgroups across both PDSA cycles limit the statistical power of subgroup analyses. Future studies should aim to collect data over a longer period to increase sample size and robustness of findings.

Third, due to the nature of this quality improvement study and the absence of ethics approval for systematic complication tracking, we could not prospectively collect data on apheresis‐related complications, including electrolyte disturbances (e.g., hypocalcemia, hypomagnesemia), coagulopathy, or volume overload symptoms such as dyspnea or peripheral edema. Although our nursing team did not observe any difference in complication rates before and after the intervention and no major adverse events were anecdotally reported during the study, the lack of prospectively collected safety data limits our ability to fully evaluate the clinical risks associated with increased total blood volume (TBV) processing. Additionally, no formal feedback assessment from staff or patients was performed, relying instead on informal feedback to gauge satisfaction with the changes implemented. Future quality improvement efforts should incorporate structured monitoring of procedure‐related complications and formal staff and patient feedback assessments to ensure patient safety and satisfaction alongside operational efficiency.

Additionally, hematologic measures such as hematocrit and platelet count before and after apheresis were not assessed. Monitoring these values could help evaluate the impact of increasing TBV processing on blood loss and collection‐related cytopenias and inform transfusion thresholds. Including this data in future studies may provide important insights into optimizing both the safety and quality of HSC collections.

Finally, the scope of balancing measures was limited. While ANC and platelet engraftment times were evaluated, long‐term patient outcomes, such as progression‐free survival, were not included in this study. Future investigations may be warranted to determine whether these interventions have any impact on long‐term transplant success rates.

## 6 Conclusions

In summary, this quality improvement initiative has led to sustained improvements in apheresis efficiency, reduced costs, and enhanced patient experience, all while maintaining high standards of care. As a result, our center has permanently reduced the default booking duration for HSC collection from 3 days to two for all patients, regardless of diagnosis. Although nursing shortages have limited our ability to increase booking frequency, addressing these staffing constraints remains a priority to fully realize the operational benefits of reduced collection days.

Future directions include structured monitoring and formal evaluation of these interventions, incorporating structured feedback from patients and interdisciplinary staff—including physicians, nursing staff, and technicians. Additional strategies to further enhance efficiency may involve pre‐emptive administration of Plerixafor prior to the first day of collection, as implemented successfully at other institutions, thus potentially reducing average high‐risk MM collections to fewer than 2 days. Other considerations include optimizing timing and dosing of mobilization agents, refining patient‐specific mobilization protocols based on risk stratification, and exploring advanced predictive analytics to better tailor mobilization strategies. Overall, we anticipate our project will serve as a model for other centers aiming to optimize the efficiency and quality of apheresis services.

## Author Contributions

R.D. collected the data, framed the ideas, drafted, and critically revised the manuscript. I.E.V. completed the data analysis and drafted the manuscript. S.S. and K.S. were responsible for data analysis, the design of the protocol, and manuscript review. S.P. and A.X. critically reviewed the manuscript. U.D. critically revised the manuscript and supervised the project.

## Ethics Statement

The authors have nothing to report.

## Conflicts of Interest

The authors declare no conflicts of interest.

## Data Availability

The data that support the findings of this study are available from the corresponding author upon reasonable request.
